# HNRNPA2B1-Mediated MicroRNA-92a Upregulation and Section Acts as a Promising Noninvasive Diagnostic Biomarker in Colorectal Cancer

**DOI:** 10.3390/cancers15051367

**Published:** 2023-02-21

**Authors:** Yiling Li, Kexin Li, Xiaoying Lou, Yue Wu, Samuel Seery, Danfei Xu, Yuqing Pei, Benheng Qian, Yuxin Wu, Shuang Liang, Kui Wu, Wei Cui

**Affiliations:** 1 State Key Laboratory of Molecular Oncology, Department of Clinical Laboratory, National Cancer Center/National Clinical Research Center for Cancer/Cancer Hospital, Chinese Academy of Medical Sciences and Peking Union Medical College, Beijing 100021, China; 2 Division of Health Research, Faculty of Medicine and Health, Lancaster University, Lancaster LA1 4YG, UK; 3 Department of Laboratory Medicine, West China Hospital of Sichuan University, Chengdu 610041, China; 4 Department of Cardiology, The Second Affiliated Hospital of Wenzhou Medical University, 109 Xueyuan Road, Wenzhou 325027, China

**Keywords:** colorectal cancer, miR-92a, biomarker, hnRNPA2B1, N6-methyladenosine (m6A)

## Abstract

**Simple Summary:**

Colorectal cancer (CRC) is the second leading cause of cancer deaths, with approximately 15–25% of the primary diagnosis. MicroRNA-92a (miR-92a) is considered one of the most promising biomarkers for colorectal cancer diagnosis; however, at present the diagnostic accuracy of miR-92a for CRC remains inconclusive and the upstream regulatory mechanism is not well understood in CRC development. In this study, we firstly found that serum/plasma miR-92a had better diagnostic efficacy compared to stool samples in CRC through meta-analysis. Additionally, we confirmed that decreased miR-92a expression and secretion take place in CRC cells after knockdown of heterogeneous nuclear ribonucleoproteins A2/B1 (HNRNPA2B1) in vitro. The common peaks of N6-methyladenosine (m6A) and HNRNPA2B1 in proximate miR-92a suggested HNRNPA2B1 mediated miR-92a expression through m6A modification. Thus, we identified that miR-92a derived from blood could be a better noninvasive biomarker, and provide insight into the mechanism of miR-92a in the expression and secretion in CRC.

**Abstract:**

MicroRNA-92a (miR-92a) may serve as a novel promising biomarker in multiple cancers, including colorectal cancer (CRC); however, the diagnostic accuracy and the underlying molecular mechanism of miR-92a in CRC is poorly understood. We first carried out meta-analysis and found that serum/plasma miR-92a yield better diagnostic efficacy when compared to stool samples and CRC tissues, and this finding was validated by our independent study through stool sample. Multiple bioinformatics assay indicated that miR-92a expression was positively correlated with heterogeneous nuclear ribonucleoproteins A2/B1 (HNRNPA2B1) expression and closely related with the clinical characteristics of CRC. Experimental evidence showed that knockdown of HNRNPA2B1 could significantly decrease miR-92a expression and secretion in RKO cells. HNRNPA2B1 mediated miR-92a via m6A RNA modification. These findings indicate that HNRNPA2B1-m6A RNA modification-derived MicroRNA-92a upregulation and section from the local CRC acts a candidate noninvasive serum biomarker in colorectal cancer. Our study provides a novel insight into miR-92a mechanisms in relation to both expression and secretion for CRC diagnosis.

## 1. Introduction

Colorectal cancer (CRC) is the third most frequently diagnosed cancer. Aside from being the second leading cause of cancer deaths [1], diagnosis is traumatic and has life-changing consequences. Unfortunately, 20% of those newly diagnosed have carcinoma with propensity for metastasis, which worsens prognosis, especially in terms of 5-year survival [2]. It is noted that CRC is also an age-associated malignancy, with nearly 70% of patients older than 65 years and 40% over 75 years. Furthermore, older CRC patients are more difficult to treat and have poor performance status, because of their decreased immune function, increased risk of treatment-related toxicities, and severe postoperative complications, including frailty, sarcopenia, hypoalbuminemia, and other comorbidities [3]. A multivariable analysis showed that the overall survival (OS) was shorter among older CRC patients and those with synchronous liver metastasis at diagnosis [4]. Therefore, there is a need to develop accurate, early diagnostic methods to ensure that CRC interventions are as effective as possible. At present, colonoscopy is the gold standard diagnostic method; however, this is an invasive procedure which is not always accepted and can influence health-service-seeking behaviors. Given that postponing diagnosis will negatively affect outcomes, there is a need to explore novel methods to improve early detection and diagnosis.

MicroRNA (miRNA) is a highly conserved short noncoding RNA, which consists of approximately 18–25 nucleotides, essential for regulating post-transcriptional gene expression by mediating messenger RNA (mRNA) degradation or blocking protein translation [5]. Studies into miRNA-92a, which belong to miR-17/92 clusters, reveal that this microRNA has a pivotal role in regulating tumorigenesis, and is often overexpressed in several different types of cancers, including CRC [6]. It is widely considered that miR-92a is released by the tumor mass and can be derived from blood (including plasma and serum), or from stool samples which also contain exfoliative CRC cells. The diagnostic power of miR-92a expression suggests that it has the potential to be a noninvasive diagnostic biomarker in CRC [7]. At present, the diagnostic accuracy of miR-92a for CRC remains inconclusive because study designs vary and the related data are often incomparable, and therefore cannot be synthesized. However, there may be a statistical method to determine whether miR-92a levels in plasma/serum samples or stool samples generate a superior diagnostic performance. This is still a relatively new knowledge base, with few researchers having focused on comparing sampling methods [8].

N6-methyladenosine (m6A) is an abundant RNA modifier in eukaryotes, which positively relates to tumorigenesis and therefore progression [9]. Interactions between m6A and miRNA can regulate proliferation, metastasis, drug resistance, and antitumor immunity of cancer cells. Notably, RNA-binding proteins play a leading role in m6A modification functions [10]. For example, RNA-binding heterogeneous nuclear ribonucleoprotein A2/B1 (HNRNPA2B1) proteins can bind to sites containing m6A and have an important influence over alternative splicing. HNRNPA2B1 can also mediate m6A-dependent microRNA processes and affect miRNA production [11,12]. However, the role of HNRNPA2B1 in regulating miRNA in CRC has not been clarified; therefore, it remains necessary to investigate the regulatory mechanisms involved in the development of CRC. In this study, we systematically reviewed and the meta-analyzed evidence around miR-92a as a CRC biomarker. Synthesized data from blood samples and stool samples were compared in terms of colorectal cancer diagnosis performance. In addition, we prospectively recruited independent patients for clinical efficacy validation, focusing on HNRNPA2B1 and the expression and role of miR-92a in CRC.

## 2. Materials and Methods

### 2.1. Meta-Analysis and Statistical Analysis

PubMed, Cochrane Library, Embase, and Web of Science were searched from inception until May 2022. Search terms and eligibility criteria are shown in Appendix B. Two reviewers independently extracted data according to predefined eligibility criteria. Data abstracted included first author, publication year, region, number of patients and controls, specimen type, differential expression, and P values. In addition, the area under curve (AUC), number of true positives (TP), false positives (FP), false negatives (FN), and true negatives (TN) were extracted for blood and stool sample types combined with our own clinical dataset based on stool samples. TP, FP, FN, and TN test results were pooled to obtain pooled sensitivity, specificity, and diagnostic odds ratio (DOR) using Stata (version 15.1). Summary receiver operator characteristic (SROC) curve and AUC were generated to provide an opportunity for visual representation.

### 2.2. Recruitment of Volunteers for Prospective Studies

This study was a cross-sectional blind comparison and was approved by the Ethics Committee of Cancer Hospital, Chinese Academy of Medical Sciences (ethical approval no. 22/139-3340). All subjects signed the informed consent. We prospectively enrolled 144 outpatients undergoing colonoscopy or preoperative colorectal cancer patients for opportunistic screening from April 2021 to January 2022 in Cancer Hospital of Chinese Academy of Medical Sciences. Inclusion and exclusion criteria are further displayed in Appendix B.

### 2.3. MiRNA Extraction and qPCR in Stool Samples

Stool sample collection and the miR-92a test were conducted with a double-blind method. Total RNA (about 0.5 g of stool) was extracted using the REColonTM Nucleic Acid Extraction kit (GeneBioHealth, Shenzhen, China) and reverse-transcribed into cDNA by adding 30 ng of RNA for each sample. The qPCR was performed with a LightCycler 480 system (Roche Diagnostics, Indianapolis, IN, USA) and a standard curve was established to calculate the copy number of each sample. Samples with a copy number lower than 902 copies/μL were interpreted as negative, and those larger than or equal to 902 copies/μL were interpreted as positive.

### 2.4. Bioinformatics Analysis of CRC Datasets

The gene expression profiles of total 499 CRC and 228 normal colorectum human tissue samples were retrieved from the Gene Expression Omnibus, also known as GEO, with six HNRNPA2B1 and six miRNA expression datasets (Appendix B). Exactly matched RNA-seq and miRNA expression data were obtained from TCGA datasets with a total of 545 CRC and adjacent tissues to explore the correlation between HNRNPA2B1 and miR-92a. Clinicopathological data including TNM stage, lymph node status, and metastasis status were extracted from TCGA miRNA expression datasets. The HNRNPA2B1-targeted miRNA data were selected from the research of Lee et al. [13] and 2 GEO datasets (GSE108153 and GSE49246). For the m6A and HNRNPA2B1 enrichment peaks, we retrieved data, respectively, from the GSE29714 and GSE107768 datasets.

### 2.5. Cell Culture In Vitro

CRC cell lines (RKO, SW480 and HCT116) were purchased from the National Infrastructure of Cell Line Resources in China (Beijing, China). MEM and 1640 medium with 10% FBS (ThermoFisher Scientific, Inc., Waltham, MA, USA) were used to incubate the CRC cell lines at 37 °C with 5% CO_2_.

### 2.6. m6A RNA Methylation Assay

TRIzol (ThermoFisher Scientific, Catalog number: 15596026, Waltham, MA, USA) was used to extract total RNA from SW480. The chemically fragmented RNA was incubated with m6A antibody (Synaptic Systems, 202111, Göttingen, Germany) to immunoprecipitate according to the guideline of EpiMark N6-Methyladenosine Enrichment Kit (NEB) as we previously reported [14]. Enrichment of m6A containing mRNA was then analyzed using qRT-PCR.

### 2.7. RNA Immunoprecipitation Assay

RIP was performed with Magna RIP RNA-binding protein immunoprecipitation kits (Millipore, 17-700, Burlington, MA, USA). Antibody against HNRNPA2B1 (Proteintech 14813-1-AP, Rosemont, IL, USA) was used. Total RNA from SW480 cell line was extracted and depleted of ribosomal RNA. The RNA protein complexes were washed and mixed with RIP immunoprecipitation buffer. Finally, RNA expression was evaluated by qRT-PCR, which was normalized to input and IgG.

### 2.8. SiRNA Transfection

To knockdown endogenous gene expression, two synthesized duplex RNAi oligos (Synbio Technologies) targeting human HNRNPA2B1 mRNA sequences were used, and are provided in the Appendix B (Table A3). The transfection of small interfering RNAs (siRNA) was performed using RNAiMax Reagent (Invitrogen, Waltham, MA, USA). The cells were transfected with siRNA according to the manufacturer’s instructions, and the efficiency of siRNA transfection was evaluated by immunoblot analysis and qRT-PCR.

### 2.9. Secreting miRNA Isolation

Five mL RKO cell culture medium was collected from MEM medium with 10% exosome depleted FBS (VivaCell, Yerevan, Armenia) which was changed one day before the transfection with siRNA or siNC treatment. Then, siRNAs were transiently transfected into RKO cells for 0, 24, and 48 h, and total RNA from medium was extracted using exoEasy maxi Kit (QIAGEN, Germantown, MD, USA) to collect the secreting miRNA according to the manufacturer’s protocol.

### 2.10. RNA Extraction, Reverse Transcription, and RT-qPCR

After extracting the RNA from adherent cells, exfoliated cells and culture medium were collected for RNA extraction by TRIZOL regent as we previously reported [15], and cDNAs were synthesized with PrimeScript™ IV 1st strand cDNA Synthesis Mix (Takara, Beijing, China) for mRNA and Mir-X™ miRNA First-Strand Synthesis (Takara Bio USA, Inc., San Jose, CA, USA) for miRNA. QRT-PCR analysis was measured using the Taq Pro Universal SYBR qPCR Master Mix (Vazyme Biotech Co., Ltd., Nanjing, China). Each assay was carried out in triplicate by the Light Cycler 480 Instrument (Roche, Basel, Switzerland). The primers for RT-qPCR are provided in the Appendix B (Table A4).

### 2.11. Western Blot Analysis

Cells after treatment were ruptured with RIPA Cell Signaling Technology buffer containing Protease Inhibitor Cocktail (Thermo Fisher, Waltham, MA, USA; 78430). Approximately 40 μg of total protein was resolved by SDS–polyacrylamide gel electrophoresis, then transferred onto the polyvinylidene fluoride (PVDF) membranes. After blocking with 5% nonfat milk, the membranes were incubated overnight at 4 °C with antibodies against HNRNPA2B1 (1:200, sc-53531, Santa Cruz Biotechnology, Dallas, TX, USA) and alpha-Tublin (1:2000, t8203, Sigma, St. Louis, MO, USA). Further, mouse monoclonal antibodies were blotted at room temperature for 2 h. The bands were visualized with an enhanced chemiluminescent reagent (ThermoFisher, Waltham, MA, USA).

### 2.12. Statistical Analysis

All statistical analyses were performed by GraphPad Prism 9 (GraphPad Software, Inc.). Continuous variables were presented as means with SDs and the unpaired two-tailed Student’s *t*-test or Wilcoxon’s tests, as well as Spearman’s method, were used to compare differences between two groups with a significance of *p* < 0.05.

## 3. Results

### 3.1. MiR-92a Was Differentially Expressed among Tissue, Blood, and Stool Sample Types in CRC

**Figure 1:**
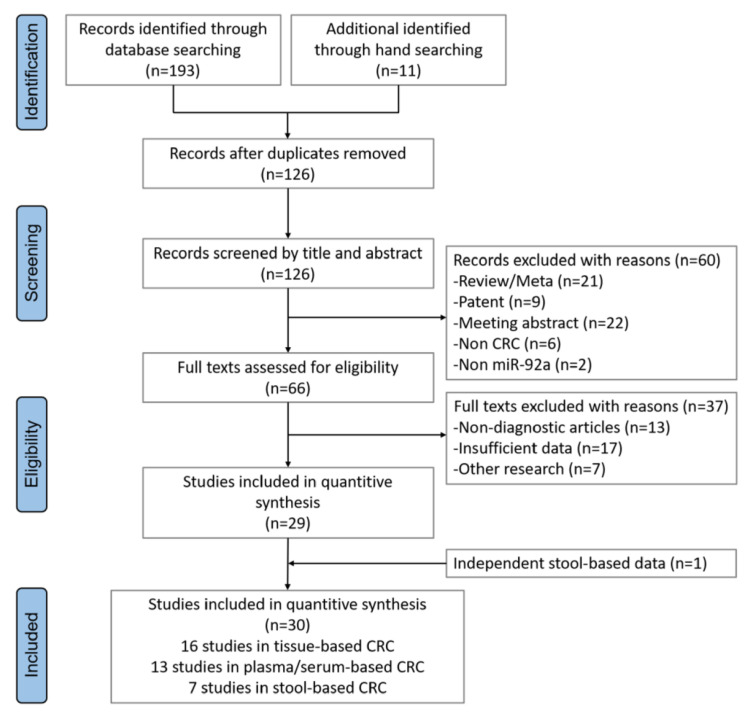
Study selection for miR-92a test in colorectal cancer (CRC).

Initially, we systematically searched and reviewed studies related to miR-92a in CRC. A total of 204 studies were retrieved from public databases and 78 duplicates were immediately excluded. After screening titles and abstracts, 66 articles were considered eligible for data extraction. After reviewing the articles in detail, 37 further studies were excluded due to lack of diagnostic data or irrelevant researches. A total of 30 studies containing only stool-based data were eventually deemed pertinent [7,8,16,17,18,19,20,21,22,23,24,25,26,27,28,29,30,31,32,33,34,35,36,37,38,39,40,41]. The flowchart presented below provides the process of inclusion/exclusion for this systematic review and meta-analysis (Figure 1). The total included studies involved 2345 CRC patients and 1954 controls. The types of specimens were classified as tissue (*n* = 16), plasma or serum (*n* = 13), and stool (*n* = 6), shown in Table 1. The weighted fold changes (FC) were 2.64 ± 1.30, 5.07 ± 3.07, and 28.42 ± 49.09, separately, for tissue, stool, and blood (including plasma and serum). This confirmed the feasibility of miR-92a as an optional diagnostic biomarker, which was highly expressed in CRC with three sampling methods, especially with blood and stool sampling.

### 3.2. Diagnostic Accuracy of miR-92a Based on Plasma/Serum Appears Better than Stool Samples

Previous studies suggested that miR-92a was highly expressed in CRC, indicating a potential effective diagnostic marker. Compared with colonoscopy and biopsy, blood or stool examination is a more convenient and effective choice for patients. However, which is better for diagnosis efficiency is still confusing. In order to explore this issue, we included a total of 19 studies (blood = 13, stool = 7) for further meta-analysis. There were 835 CRC patients and 598 controls in the plasma/serum group, and 838 CRC patients and 793 controls in the stool sample group (Table 2). It is worth mentioning that one included article provided both plasma- and stool-based miRNA data [8].

**Figure 2:**
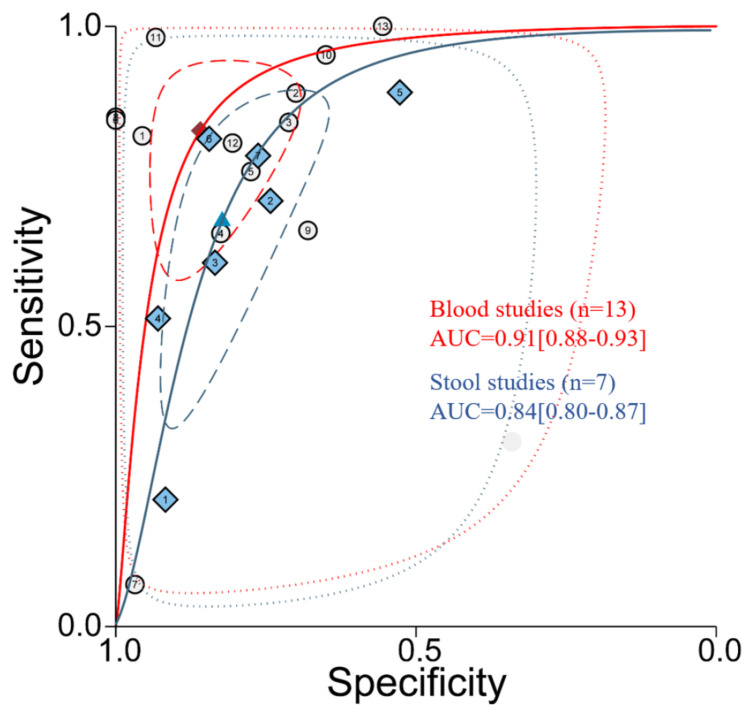
Summary receiver operating characteristic curves for miR-92a in CRC and controls. The pooled blood studies (*n* = 13) for CRC had an AUC of 0.91 (95% CI: 0.88–0.93). The pooled stool studies (*n* = 7) for CRC had an AUC of 0.83 (95% CI: 0.80–0.86). *n* means the included studies. Circles represent included blood studies, and squares represent included stool studies.

The CRC estimates for sensitivity and specificity based on the plasma/serum group were 0.83 (95% CI, 0.67–0.92) and 0.86 (95% CI, 0.75–0.92), whereas the pooled sensitivity was 0.71 (95% CI, 0.49–0.86) and specificity was 0.81 (95% CI, 0.72–0.88) in the stool group. The combined DOR was 28.98 (95% CI, 11.67–91.92) and 10.31 (95% CI, 5.63–18.88) in the plasma/serum and stool group (Table 3). The SROC curve of the blood group and the stool group was drawn using Stata 15.1, and highlighted a better diagnostic value in the blood group than the stool group, with AUC of 0.91 (0.88–0.93) vs. 0.84 (0.80–0.87). In summary, the plasma/serum miR-92a has a better diagnostic accuracy than the stool-based biomarker, especially in terms of sensitivity (Figure 2).

### 3.3. MiR-92a as a Potential Biomarker in the Independent Prospective Data from Stool Samples

**Figure 3:**
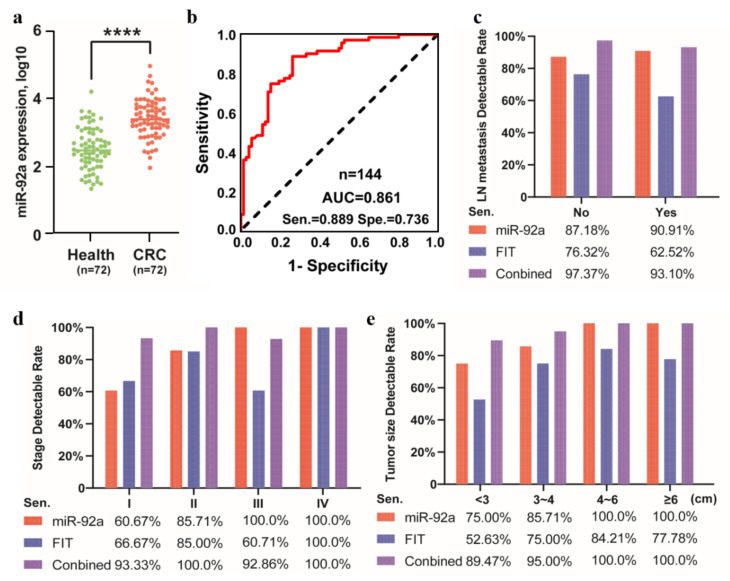
Independent stool-based data for recruited independent patients. (**a**) Comparison of levels of miR-92a from CRC patients and health controls; (**b**) ROC analysis using miR-92 to discriminate CRC; (**c**–**e**) The detection rate of miR-92a compared across CRC lymph node metastasis, stage, and tumor size, respectively. **** *p* < 0.0001.

To further verify the clinical efficacy of miR-92a as a diagnostic biomarker, we recruited a total of 144 volunteers for opportunistic screening (CRC = 72, healthy individuals = 72), including 82 males and 62 females. The demographic and clinicopathological characteristics of the enrolled CRC patients and healthy volunteers are displayed in Appendix A, Table A1 and Table A2. Compared to the normal group, the miR-92a expression was significantly increased in colorectal cancer group with 1.36-fold change (Figure 3a). The diagnostic efficiency of miR-92a test in CRC patients was evaluated through ROC analysis, and the AUC was 0.861 (95% CI: 0.801–0.901, *p* < 0.05) with a sensitivity/specificity of 0.889/0.736, suggesting that this noninvasive detection method had a desired diagnostic ability for colorectal cancer (Figure 3b). Next, as shown in the histograms, the positive detection rate of miR-92a also increased with lymph node metastasis (Figure 3c), tumor stage development (Figure 3d), and tumor size increasing (Figure 3e). In summary, with the progression and metastasis of colorectal cancer, the differential expression of miR-92a becomes higher, indicating that miR-92a could be a promising biomarker for CRC.

### 3.4. HNRNPA2B1 May Regulate miR-92a through m6A Modification in Colorectal Cancer

**Figure 4:**
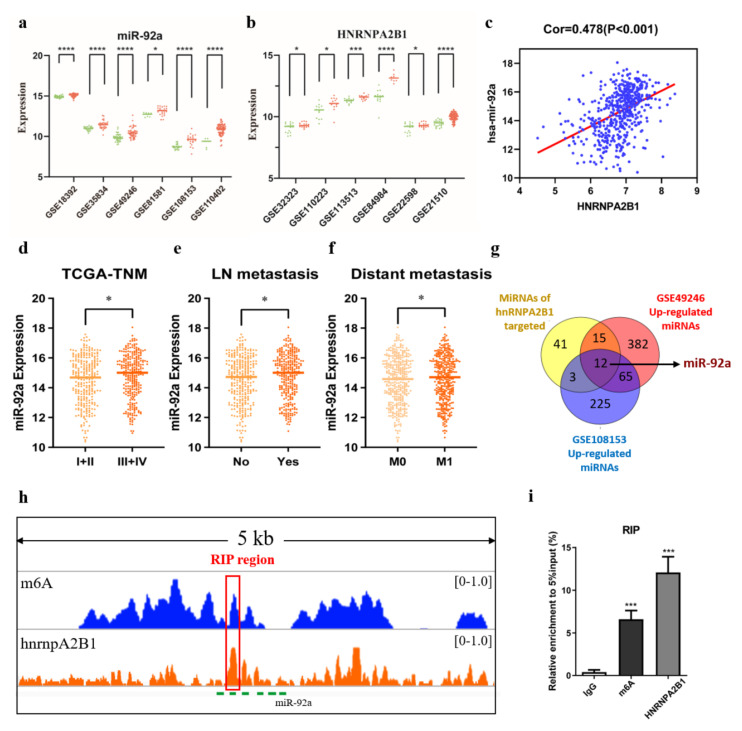
HNRNPA2B1 may regulate miR-92a expression through m6A modification. (**a**,**b**) Higher miR-92a and higher HNRNPA2B1 expression in a series of GEO datasets; (**c**) positive correlations between HNRNPA2B1 and miR-92a; (**d**–**f**) the relationship between miR-92a expression and clinical characteristics of TNM stage, lymph node metastasis, and distant metastasis, respectively; (**g**) the Venn diagram of HNRNPA2B1-targeted miR-92a from the GEO datasets (GSE108153, GSE49246) and Lee’s study; (**h**) the enrichment peak of m6A and HNRNPA2B1 in miR-92a sites from the HITS CLIP sequence; (**i**) RIP-qPCR showed that the m6A and HNRNPA2B1 peak region was occupied by the nearby miR-92a sites in SW480 cells. * *p* < 0.05; *** *p* < 0.001; **** *p* < 0.0001.

Further, we would like to investigate the reason for the elevated miR92a based on blood and stool samples in CRC, so as to better understand the mechanisms. Recently, Alarcón et al. [11] reported that HNRNPA2B1 could affect the production of miRNAs by mediating primary microRNA processing. Thus, we hypothesized that HNRNPA2B1 might be involved in the progression of CRC by regulating miR-92a expression. To explore the mechanism of m6A regulator HNRNPA2B1 and miR-92a in CRC, we first analyzed the expression profiling of m6A reader HNRNPA2B1 in six GEO datasets with 193 CRC and 100 normal colorectal tissues, showing that HNRNPA2B1 was highly expressed in CRC patients, and a similar result was displayed in miR-92a expression from six other GEO datasets (CRC = 306, the normal = 128), shown in Figure 4a,b. Next, strong positive correlations between HNRNPA2B1 and miR-92a were identified in the TCGA dataset (R = 0.352, *p* < 0.001, Figure 4c). Similar to the results of the validated stool samples, miR-92a expression from the TCGA database also had the higher expression with tumor stage development (Figure 4d), lymph node metastasis (Figure 4e), and distant metastasis (Figure 4f).

Notably, 12 miRNAs overlapped by increasing miRNAs in CRC (GSE108153 and GSE49246) and HNRNPA2B1-targeted miRNAs (Lee’s study), which included miR-92a (Figure 4g). The enrichment peak of m6A and HNRNPA2B1 from GSE29714 and GSE107768 demonstrated the colocalization of HNRNPA2B1 and m6A modification nearby miR-92a sites (Figure 4h). To address whether HNRNPA2B1 regulates miR-92a expression through m6A modification, in vitro experiments were employed via m6A RNA methylation tests and RNA immunoprecipitation tests. Through RIP-qPCR analysis, the Figure 4i materialized m6A and HNRNPA2B1 could occupy the miR-92a nearby sites, consistent with the HITS CLIP-seq results.

### 3.5. HNRNPA2B1 May Regulate the Expression and Secretion of miR-92a In Vitro

**Figure 5:**
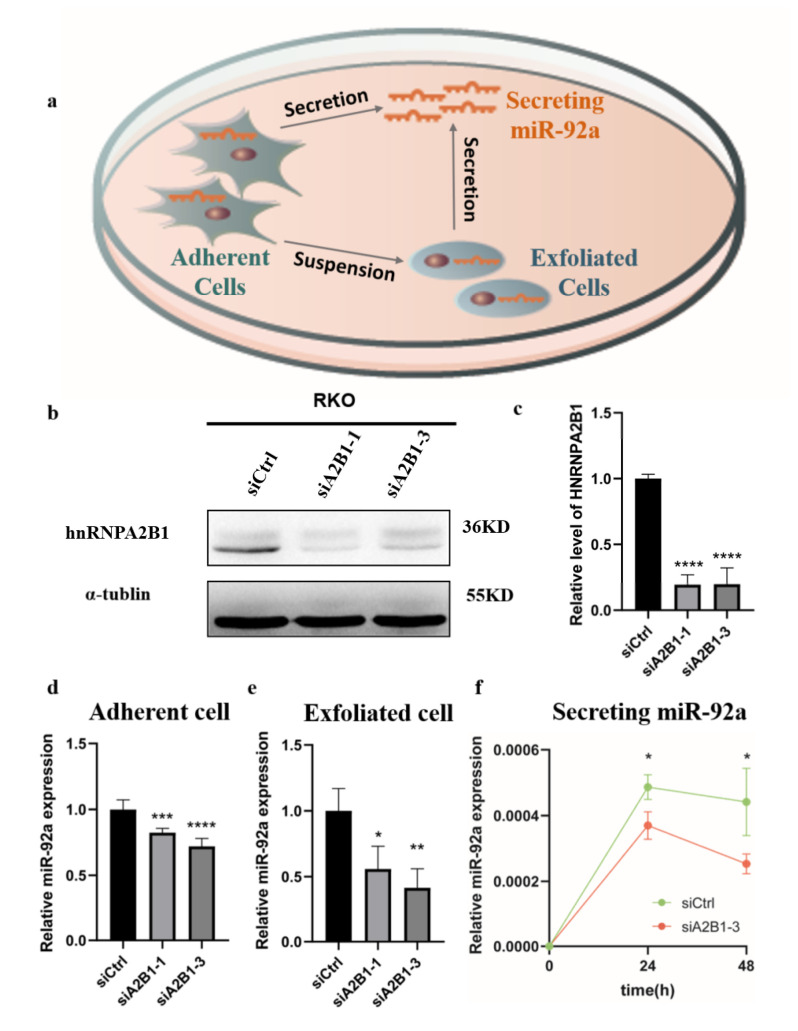
In vitro experiments confirmed that HNRNPA2B1 may regulate the expression and secretion of miR-92a. (**a**) Pattern diagram: the adherent cells, exfoliated cells, and secreting miR-92a in the culture dish were detected, respectively, for RKO cell line; (**b**) HNRNPA2B1 expression was successfully knocked down by si-HNRNPA2B1 transfection for 48 h in RKO cells; (**c**) HNRNPA2B1 expression in adherent CRC cells with or without HNRNPA2B1 knockdown was measured by qRT-PCR (*n* = 3); (**d**–**f**) miR-92a expression from adherent cells, exfoliated cells, and secreting in culture medium with or without HNRNPA2B1 knockdown (*n* = 3), respectively. * *p* < 0.05; ** *p* < 0.01; *** *p* < 0.001; **** *p* < 0.0001.

**Figure A1:**
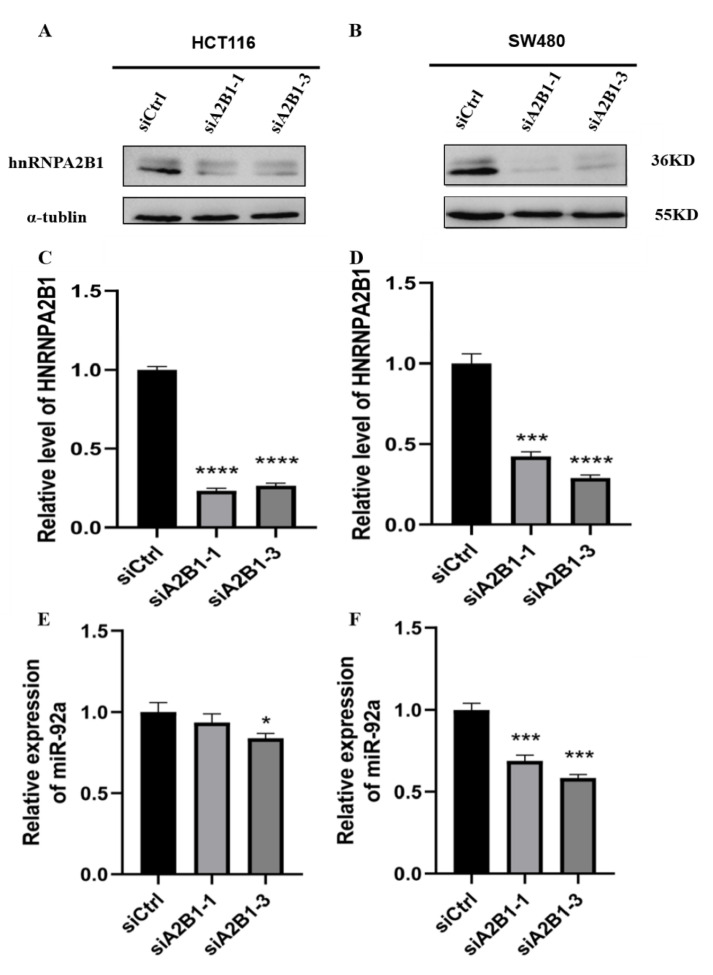
Knockdown experiments confirmed that HNRNPA2B1 may regulate the expression of miR-92a. (**A**,**B**) HNRNPA2B1 expression was successfully knocked down by si-HNRNPA2B1 transfection for 48 h in HCT116 and SW480 cells; (**C**,**D**) HNRNPA2B1 expression in HCT116 and SW480 cells with or without HNRNPA2B1 knockdown was measured by qRT-PCR (*n* = 3); (**E**,**F**) miR-92a expression in HCT116 and SW480 cells with or without HNRNPA2B1 knockdown (*n* = 3), respectively. * *p* < 0.05; *** *p* < 0.001; **** *p* < 0.0001.

To the best of our knowledge, miR-92a was produced by the sites of tumor tissue, and then exfoliated into the intestinal tract or secreted into the blood circulation system. Our previous finding demonstrated that the expression of miR-92a in CRC from tissue, stool, and blood was extremely high and miR-92a co-immunoprecipitate was regulated by HNRNPA2B1. In this way, we next wondered whether HNRNPA2B1 could mediate the expression and secretion of miR-92a in CRC, through in vitro experiments. As Figure 5a displays, the model diagram mimicked the process of miR-92a shedding from tumor tissue (adherent cells) into the intestine by detecting MiR-92a expression in stool samples (exfoliated cells), and the process of entering the bloodstream through the circulatory system (secreting RNA). Firstly, we established transient HNRNPA2B1 knockdown models in RKO cells with the siRNA sequence. Figure 5b,c evidences the successful knockdown of HNRNPA2B1, respectively, in protein and mRNA levels in RKO cells. Similar results in HCT116 and SW480 cell lines are shown in Figure A1. Then, we compared the expression of miR-92a in RKO adherent and exfoliated cells. The results confirmed that miR-92a was significantly downregulated in CRC with the knockdown of HNRNPA2B1 in protein and RNA level (Figure 5d,e). Finally, after the treatment of RKO cells at different time periods (0 h, 24 h, and 48 h), it was found that the secretion of miR-92a was significantly reduced after knockdown of HNRNPA2B1 (Figure 5f), indicating that HNRNPA2B1 may regulate the secretion of miR-92a by controlling the expression of miR-92a.

## 4. Discussion

Numerous primary and secondary studies have found that early detection and diagnosis will decrease CRC-specific mortality compared with no screening [43]. However, due to invasiveness, associated costs, and stigma, colonoscopy is often refused by patients, and postponing this investigation can have a devastating impact on outcomes once diagnosed. The public may be more willing to endure less invasive sampling methods such as blood and stool collections. Therefore, we need to ensure we are using the most effective, least invasive technique for tumor screening. As routine screening methods, guaiac-based fecal occult blood tests (gFOBT), fecal immunochemical test (FIT), and serum-based SEPT9 DNA methylation test (Epi proColon) have relatively low sensitivities for CRC [44]. Additionally, the current multitarget stool DNA test is not considered cost-efficient, and there is a lack of evidence from large-scale prospective studies. Furthermore, colorectal cancer is a highly heterogeneous clinical entity; genetic KRAS, NRAS, BRAF, or HER2 genes also work as the critical diagnostic and prognostic biomarkers. MiRNAs regulate critical pathways involved in the CRC pathogenesis, including the p53, PI3K, RAS, MAPK, and EMT transcription factors, and Wnt/β-catenin pathways. MiRNAs could be a further therapeutic potential to explore effective targeting of KRAS-mutant CRC [45]. Therefore, it is important to consider diagnostic biomarkers, especially those associated with tumorigenesis, such as microRNA, as they may provide insight into multiplicity and proliferation.

Lin et al. summarized human genome regulation and carcinogenesis diversity which are closely associated with miRNAs from tissue sample source [46]. For plasma/serum samples, through the systemic circulatory system, miRNAs are secreted or shed from the local tumor tissue and transported to the blood packed in exosomes or combined with biomacromolecules [47,48]. Regarding stool samples, miRNAs are directly derived from exfoliated colonocytes through the intestinal cavity [49]. MiRNAs extracted from stool samples are easy to obtain from patients, and have been demonstrated to have high quality [50]. Therefore, several researchers have suggested that miRNAs based on blood or stool have potential as diagnostic biomarkers, specifically for CRC. However, even though several studies have reported the efficacy of miR-92a as a CRC diagnostic biomarker, no one has focused on systematically analyzing miR-92a derived from blood or stool samples in order to determine which one can better assess the risk of colorectal cancer.

We firstly synthesized evidence and compared the miR-92a diagnostic value for colorectal cancer based on plasma/serum and stool samples. We found that the diagnostic efficiency of miR-92a based on blood samples appears better compared to stool samples. Interestingly, there was no diagnostic efficiency difference of miR-92a expression level between plasma and serum samples through meta-regression analysis (data not shown). We were also able to validate that miR-92a could be used to distinguish colorectal cancer patients from controls, yielding an AUC of 0.861 with pooled sensitivity of 89%. However, pooled specificity dropped to around 74% in the prospective element of this study when independently verifying with external data, whereas under meta-analysis, we found that the stool sample group had an AUC of 0.847 with an 84% specificity. However, this time, sensitivity dropped to 68%, which needs to be considered in more detail. For example, the stool itself may be more heterogeneous and more difficult to standardize across a small sample of individuals. As described previously, sampling different parts of the stool itself is also likely to influence the within-specimen variations [51]. Additionally, for stool samples, it is not always possible to have exact exposure times when exfoliated cancer cells fall from the colorectum. This may result in a number of surviving cells being reduced in the stool. In addition, compared with phlebotomy, which is conducted in clinics, stool specimens tend to be prepared at home and unaided, which adds to the number of potentially confounding factors.

Since blood- and stool-derived miR-92a has been identified as a promising biomarker for CRC, we further investigated the upstream regulatory mechanisms of miR-92a. This was performed to better understand the underlying mechanisms involved in miR-92a elevation. Previous research has shown that HNRNPA2B1 could interact with microRNA and its complex protein DGCR8 to promote the miRNA processing through binding with the m6A mark [11]. In addition to this, Villarroya et al. found that HNRNPA2B1 can control miRNA sorting through binding to the sumoylated special motifs [52]. It is worth mentioning that O’Grady et al. considered that the regulation of RNA profile in the cell was generally thought of as a balance between transcription and decay. They found that HNRNPA2B1 might regulate the export the secreting miRNA to control the intracellular abundance [53]. The regulatory mechanisms of miR-92a on HNRNPA2B1 are varied, and it is therefore important that we focus on gaining insights into these mechanisms of action.

As one of the most important epigenetic modifiers in eukaryotes, m6A modification may affect miRNA splicing and carcinogenic maturation [54]. Researchers have also previously found that loss of methyltransferase, such as METTL3, causes downregulation of m6A modification levels for miR-100 and miR-125b. This process thereby inhibits miRNA expression and possibly causes specific drug resistances [55]. Yue et al. found that miR-96 promotes the initiation and progression of colorectal cancer by modulating the AMPKα2-FTO-m6A/MYC axis, which again highlights the underlying mechanisms involved in CRC development [56]. Regarding the m6A reader, IGF2BP2 interfered with RAF-1 degradation via miR-195 and promoted proliferation and survival of CRC cells [57]. Additionally, miR-6125 knockdown is thought to promote CRC proliferation through YTHDF2-dependent recognition of m6A-modified GSK3β [58]. Taken together, these findings provide evidence that there is an interaction between m6A modification and miRNA in CRC development. Combined with the mechanism of HNRNPA2B1 in the regulation of miRNA secretion, we speculate that HNRNPA2B1 may regulate the expression of miR-92a through m6a modification in colorectal cancer.

In vitro experiments illustrate that the expression level of miR-92a significantly decreased after knockdown of HNRNPA2B1. In addition, we closely monitored miR-92a in adherent cells, exfoliated cells, and those secreted from cell culture mediums for the expression level of miR-92a based on tissues, stool, and blood sample types, and we found that HNRNPA2B1 did regulate the expression and secretion of miR-92a. Further, RIP-qPCR experiments confirmed the existence of HNRNPA2B1 and m6A-modified sites adjacent to miR-92a. These results infer that HNRNPA2B1 may be enriched near miR-92a sites through m6A recognition, so as to regulate the expression and secretion of miR-92a. Taken together, these results contribute to our understanding of heterogeneous diagnostic miR-92a-based efficacy. This research also garners insight into the regulatory mechanisms involved in HNRNPA2B1 and m6A on miR-92a in CRC.

## 5. Conclusions

In summary, we initially conducted a diagnostic meta-analysis to compare plasma/serum-based miR-92a with stool-based samples, drawing the conclusion that miR-92a from blood sources has superior diagnostic efficacy at distinguishing CRC patients from healthy controls. The expression and secretion of miR-92a appears to be regulated by HNRNPA2B1 through m6A modification, which ultimately helps to individualize colorectal cancer diagnosis and care.

## Figures and Tables

**Table 1:** MiR-92a expression in colorectal cancer (CRC) versus control.

Sample Source	Author	Year	Region	Case/Control	Fold Change	*p*
Tissue				744/635	2.64 ± 1.30	<0.0001
	Ng et al. [18]	2009	China	5/5	2.58	<0.05
	Earle et al. [30]	2010	USA	55/55	1.75	<0.0001
	Koga et al. [20]	2010	Japan	31/31	1.81	0.01
	Ma et al. [31]	2011	China	12/12	1.17	<0.05
	Wu et al. [21]	2011	China	40/40	2.18	<0.0001
	Wang et al. [23]	2012	China	57/15	2	<0.0001
	Nishida et al. [32]	2012	Japan	13/4	2.8842	<0.0001
	Ragusa et al. [33]	2012	Italy	22/5	up ^1^	<0.05
	Neerincx et al. [34]	2015	Holland	40/23	1.866	<0.0001
	Al-Sheikh et al. [35]	2016	Saudi Arabia	20/20	2.6	<0.01
	Jepsen et al. [36]	2016	Denmark	9/3	1.68	0.002
	Uratani et al. [37]	2016	USA	19/20	up	<0.001
	Slattery et al. [38]	2018	USA	217/217	2.95	0.0009
	Chang et al. [8]	2016	China	62/62	1.9	<0.001
	Brînzan et al. [28]	2020	Romania	82/82	2.32	<0.001
	Fellizar et al. [40]	2022	Philippines	41/41	7.41	<0.001
**Stool**				**766/721**	**5.07 ± 3.07**	**<0.0001**
	Koga et al. [20]	2010	Japan	197/119	8.3	<0.0001
	Wu et al. [21]	2011	China	88/101	up	<0.0001
	Chang et al. [8]	2016	China	62/62	3.48	<0.0001
	Xue et al. [24]	2016	China	50/50	1.08	<0.01
	Choi et al. [26]	2019	Korea	29/29	2.12	0.001
	Xu et al. [42]	2022	China	340/360	1.33	<0.0001
**Blood**				**835/598**	**28.42 ± 49.09**	**<0.0001**
	Ng et al. [18]	2009	China	5/5	4.45	<0.05
	Huang et al. [19]	2010	China	100/59	up	<0.0001
	Giráldez et al. [16]	2013	Spain	21/20	1.83	0.0437
	Liu et al. [22]	2013	China	200/80	up	<0.05
	Chang et al. [8]	2016	China	62/62	2.85	<0.0001
	Elshafei et al. [25]	2017	Egypt	64/27	3.38	<0.0001
	Luo et al. [27]	2019	China	57/125	up	0.007
	Shi et al. [7]	2020	China	148/68	up	<0.001
	Hassan et al. [17]	2020	Egypt	33/30	158.83	<0.05
	Elaguizy et al. [29]	2020	Egypt	50/50	1.879	0.003
	Zaki et al. [39]	2022	Egypt	54/15	62.2	<0.001
	Fellizar et al. [40]	2022	Philippines	36/36	2.5	<0.001
	Kim et al. [41]	2022	Korea	5/21	up	<0.001

^1^ Note: up means it could not point to the exact fold change number but still showed the higher expression in CRC.

**Table 2:** Characteristics of included blood and stool studies in the meta-analysis.

Sample Source	Author	Year	Specimen	Case/Control	AUC
Blood				920/650	0.910
	Ng et al. [18]	2009	plasma	90/50	0.885
	Huang et al. [19]	2010	plasma	100/59	0.838
	Giráldez et al. [16]	2013	plasma	21/20	0.857
	Liu et al. [22]	2013	serum	200/80	0.786
	Chang et al. [8]	2016	plasma	62/62	0.833
	Elshafei et al. [25]	2017	serum	64/27	0.844
	Luo et al. [27]	2019	plasma	57/125	0.603
	Shi et al. [7]	2020	serum	148/68	0.914
	Hassan et al. [17]	2020	plasma	33/37	0.887
	Elaguizy et al. [29]	2020	serum	50/50	0.672
	Zaki et al. [39]	2022	plasma	54/15	0.994
	Fellizar et al. [40]	2022	plasma	36/36	0.760
	Kim et al. [41]	2022	plasma	5/21	0.895
**Stool**				**838/793**	**0.840**
	Koga et al. [20]	2010	stool	197/119	NA
	Wu et al. [21]	2011	stool	88/101	0.780
	Chang et al. [8]	2016	stool	62/62	0.739
	Xue et al. [24]	2016	stool	50/50	0.789
	Choi et al. [26]	2019	stool	29/29	0.760
	Xu et al. [42]	2022	stool	340/360	0.870
	Li et al.	2022	stool	72/72	0.861

**Table 3:** Pooled diagnostic performance of miR-92a based on blood and stool sample types.

Studies	Pooled Sensitivity (95% CI)	Pooled Specificity (95% CI)	Pooled Odds Ratio (95% CI)
Blood	0.83 (0.67–0.92)	0.86 (0.75–0.92)	28.98 (11.67–71.92)
Stool	0.68 (0.48–0.83)	0.81 (0.72–0.88)	9.34 (5.34–16.34)

## Data Availability

The transcriptomic and clinical data of colorectal cancer were downloaded from the TCGA database (https://portal.gdc.cancer.gov/, accessed on 22 December 2022) and GEO database (https://www.ncbi.nlm.nih.gov/gds/, accessed on 22 December 2022). The stool-sample data presented in this study are available on request from the corresponding author, which are not publicly available due to privacy reasons.

## Appendix A

**Table A1:** Demographic characteristics of the enrolled CRC patients and healthy volunteers.

Characteristics		Health	CRC
Age	Mean ± SD	50.46 ± 13.68	59.90 ± 11.00
Sex	Male	35	47
	Female	37	25
Total		72	72

**Table A2:** Clinicopathological characteristics of CRC patients.

Characteristics	CRC		
	No. of Cases	miR-92a Positive (n)	Sensitivity (%)
Age, year			
Mean ± SD	59.90 ± 11.00		
Sex, *n* (%)			
Male	47	38	80.85
Female	25	20	80.00
Family history of cancers			
Yes	18	17	94.44
No/unknown	54	41	75.93
Histological type			
Adenocarcinoma	54	42	77.78
Mucinous	7	6	85.71
Others	11	10	90.91
Location			
left	9	7	77.78
right	63	51	80.95
LN metastasis			
Yes	33	28	84.85
No/unknown	39	30	76.92
Stage			
I & II	36	27	75.00
III & IV	36	31	86.11
Differentiation			
High	2	1	50.00
Moderate	46	38	82.61
Poor	18	15	83.33
Unknown	6	4	66.67

## Appendix B. Materials and Methods

### Appendix B.1. Search Terms

PubMed, Cochrane Library, Embase, and Web of Science were searched from inception until May 2022. Search terms were as follows: (1) colon OR colonic OR rectal OR rectum OR colorectal; (2) cancer OR tumor OR tumour OR carcinoma OR neoplasm OR carcinomata; (3) miR-92 OR microRNA-92 OR hsa-mir-92 OR miR-92a OR microRNA-92a OR hsa-miR-92a OR miR-9.2; (4) sensitiv* OR (sensitivity and specificity) OR (predictive AND value*) OR (predictive value of tests) OR accuracy*. Manual searching took place to identify other relevant medical articles. And especially, Xu et al. for the stool sample study is not published yet but matches the eligibility criteria [58].

### Appendix B.2. Eligibility Criteria

Selection criteria were established for screening retrieved publications. Inclusion criteria were as follows: (1) CRC studies focusing on RNA related issues; (2) sample sources for the study were based on tissue, stool, or serum/plasma; (3) control was gold standard colonoscopy or pathological diagnosis; (4) sufficient data to calculate sensitivities and specificities or to construct 2 × 2 tables for blood and stool studies.

Exclusion criteria included (1) duplicates; (2) studies which did not report miR-92a and CRC from nonhuman samples; (3) traditional reviews, meta-analyses, patents, conference/meetings abstracts, and non-full-text articles; as well as (4) studies with insufficient data or other studies for further analysis.

### Appendix B.3. Inclusion and Exclusion Criteria

Patient inclusion criteria included (1) those aged through 18 to 75; (2) patients undergoing colonoscopy or pathological examination. Exclusion criteria included (1) watery or contaminated stool; (2) participants who had recently been prescribed pharmaceutical interventions.

### Appendix B.4. GEO Datasets

**Table A3:** GEO datasets.

HNRNPA2B1 GEO_ID	miR-92a GEO_ID
GSE32323	GSE18392
GSE110223	GSE35834
GSE113513	GSE49246
GSE84984	GSE81581
GSE22598	GSE108153
GSE21510	GSE110402

### Appendix B.5. SiRNA and RT-qPCR Primers

**Table A4:** siRNA and RT-qPCR primers.

Gene	Sense (5′-3′)
hs-HNRNPA2B1-si-1	GGACCAGGAAGUAACUUUAdTdT
	UAAAGUUACUUCCUGGUCCdTdT
hs-HNRNPA2B1-si-3	GGCUUUGUCUAGACAAGAAdTdT
	UUCUUGUCUAGACAAAGCCdTdT
GAPDH-Fwd	TGACAACTTTGGTATCGTGGAAGG
GAPDH-Rev	AGGGATGATGTTCTGGAGAGCC
hnRNPA2B1-Fwd	ATTGATGGGAGAGTAGTTGAGCC
hnRNPA2B1-Rev	AATTCCGCCAACAAACAGCTT
miR-92-Fwd	TATTGCACTTGTCCCGGCCTG
Universal-Rev	GTGCAGGGTCCGAGGT
m6A-RIP-Fwd	AAGGCACTTGTAGCATTATG
m6A-RIP-Rev	CCAGAAGGAGCACTTAGG
18S-Fwd	GTAACCCGTTGAACCCCATT
18S-Rev	CCATCCAATCGGTAGTAGCG
U6-Fwd	CTCGCTTCGGCAGCACA
U6-Rev	AACGCTTCACGAATTTGCGT
